# A Negative Regulatory Loop between MicroRNA and Hox Gene Controls Posterior Identities in *Caenorhabditis elegans*

**DOI:** 10.1371/journal.pgen.1001089

**Published:** 2010-09-02

**Authors:** Zhongying Zhao, Thomas J. Boyle, Zongzhi Liu, John I. Murray, William B. Wood, Robert H. Waterston

**Affiliations:** 1 Department of Genome Sciences, University of Washington, Seattle, Washington, United States of America; 2 Department of Molecular, Cellular and Developmental Biology, University of Colorado, Boulder, Colorado, United States of America; Harvard University, United States of America

## Abstract

MicroRNAs (miRNAs) have been found to regulate gene expression across eukaryotic species, but the function of most miRNA genes remains unknown. Here we describe how the analysis of the expression patterns of a well-conserved miRNA gene, *mir-57*, at cellular resolution for every minute during early development of *Caenorhabditis elegans* provided key insights in understanding its function. Remarkably, *mir-57* expression shows strong positional bias but little tissue specificity, a pattern reminiscent of Hox gene function. Despite the minor defects produced by a loss of function mutation, overexpression of *mir-57* causes dramatic posterior defects, which also mimic the phenotypes of mutant alleles of a posterior Hox gene, *nob-1*, an Abd homolog. More importantly, *nob-1* expression is found in the same two posterior AB sublineages as those expressing *mir-57* but with an earlier onset. Intriguingly, *nob-1* functions as an activator for *mir-57* expression; it is also a direct target of *mir-*57. In agreement with this, loss of *mir-57* function partially rescues the *nob-1* allele defects, indicating a negative feedback regulatory loop between the miRNA and Hox gene to provide positional cues. Given the conservation of the miRNA and Hox gene, the regulatory mechanism might be broadly used across species. The strategy used here to explore *mir-57* function provides a path to dissect the regulatory relationship between genes.

## Author Summary

miRNAs are small RNAs found in many multi-cellular species that inhibit gene expression. Many of them play important roles in cancer and cell fate determination, but the function of most miRNAs is uncertain. Using live cell imaging and automated expression analysis, we found a miRNA gene, *mir-57*, is expressed in a position rather than tissue dependent way. Hox genes also regulate cell fate patterning along anterior-posterior (a-p) axis across different tissues. By investigating interactions between genes of these classes expressed in *mir-57* expressing cells, we demonstrated by both genetic analysis and gene expression assays that a negative feedback loop between a posterior Hox gene, *nob-1*, and *mir-57* regulates posterior cell fate determination in *C. elegans*. On the one hand, the Hox gene is required for normal activation of *mir-57* expression, and on the other, the Hox gene functions as a direct target of and is repressed by the miRNA. Given the conservation of the two genes, a negative feedback loop between Hox and miRNA genes might be broadly used across species to regulate cell fate along the a-p axis. Detailed expression analysis may provide a general way to dissect the regulatory role of miRNAs.

## Introduction

miRNAs are small endogenous RNA molecules found in most eukaryotic species that are involved in post-transcriptional regulation of genes required for cell fate determination, metabolism and carcinogenesis among other processes [1]. Like protein coding genes, miRNA genes are transcribed by RNA polymerase II [2], [3], suggesting a transcriptional regulatory mechanism similar to that of messenger RNAs. Over 155 miRNAs have been identified in *C. elegans* through a combination of molecular and bioinformatics methods [4]–[8]. Many of these are well conserved across eukaryotes, but only a few have been functionally characterized. For example in *C. elegans*, the founding members of miRNAs *lin-4* and *let-7* [9], [10] regulate *lin-14* and *hbl-1* respectively and are involved in controlling timing of development. In addition, the *lys-6* and *mir-273* miRNAs function sequentially and asymmetrically to control chemosensory neuronal development [11], [12]. *mir-61* has been shown to be a direct transcriptional target of LIN-12, and is involved in down regulation of *vav-1*, which in turn promotes LIN-12 activity in presumptive 2° VPCs [13]. *let-7* and its paralog *mir-84* act synergistically to direct cessation of molting via the conserved nuclear hormone receptors NHR-23 and NHR-25 [14]. However, most miRNA genes in *C. elegans* show no or only very subtle phenotypic effects when the gene is deleted from the genome [15], making their function elusive by classical genetic assays.

One approach to determining the function of these miRNAs would be to use their detailed expression patterns to find genes with which they might interact. We have recently developed technology that allows automated determination of embryonic expression patterns of individual cells with one minute time resolution [16], [17]. To apply the system to miRNAs and to see how the information might yield insights into their function, we examined the expression patterns of several miRNA genes of unknown function and found that *mir-57*, a miRNA gene conserved from nematodes to mammals (where it is named miR-10)[7], produced a particularly intriguing expression pattern. Strains carrying the *mir-57* promoter fused to a red fluorescent reporter, mCherry, showed the gene is exclusively expressed in the posterior sublineages of ABp(l/r)(a/p)p and a variety of sublineages of the C founder cell. These lineages produce a variety of cell types but have in common a posterior location in the embryo. Genetic analysis of a deletion mutant and overexpressing lines of *mir-57* supported its role in the development of the tail of the animal. This apparent position rather than tissue/cell dependent expression pattern and impact led us to examine its interactions with other genes known to be involved in posterior fate specification, including *nob-1, vab-7*, and members of the *Wnt* and *Notch* pathways, *pop-1* and *lag-1*. Interactions of *mir-57* and *nob-1* mutants along with the presence of a putative *mir-57* binding site in the 3′ UTR of a *nob-1* isoform suggested that *mir-57* might directly regulate *nob-1* expression. To test this we examined the effects of the *nob-1* 3′ UTR on reporter expression. Our combined results from functional assays provide support for a negative regulatory loop between the miRNA and Hox gene, giving *mir-57* an important role in posterior fate determination.

## Results

### *mir-57* is expressed in posterior cells across tissue types

**Figure 1:**
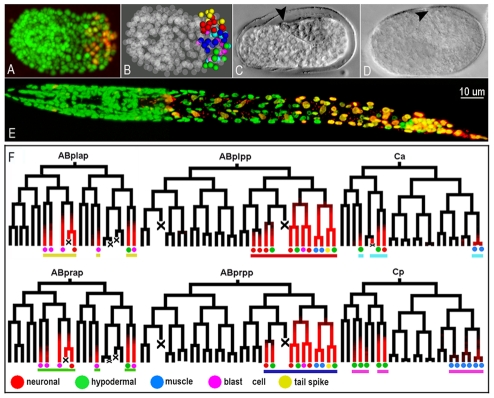
The *mir-57* gene is expressed in posterior sublineages in the posterior regions of the animal. (A) A 3-D projection of a confocal z-stack of a comma stage embryo with a stably integrated mCherry reporter driven by a *mir-57* promoter segment (p*mir-57*::HIS-24::mCherry). The *mir-57* reporter (red, or yellow when co-localized with green) is detected only in the posterior cells of the embryo. The embryo is shown in a ventral view with anterior to the left. The embryo is also ubiquitously labeled with GFP for lineaging. (B) A space filling model of the same embryo with expressing cells color coded by their lineage origins (see panel F for color code). Non-expressing cells are gray and rendered partially transparent. Note the *mir-57* expressing cells are clustered within the posterior region of embryo. (C) *In situ* hybridization of a one-and-a half fold wild type (N2) embryo using a DIG labeled anti-sense LNA *mir-57* probe (See Materials and Methods). The anti-DIG staining is apparent as darker regions over the tail (arrowhead). (D) The same *in situ* staining for *mir-57(gk175)* deletion embryos. No apparent staining was seen in the tail region (arrowhead). (E) A 3-D projection of an L1 larva expressing both *mir-57* (red) and the lineaging marker (green). Most expressing cells are seen in the tail of the animal except for a few intestinal cells. (F) Embryonic expression of *mir-*57 within cell lineages. Expressing cells were detected in the four AB sublineages and the C sublineages beginning from the 200 cell stage. The fates of the expressing cells are color coded below the leaves. The color coding of bars under each sublineage correspond to those used in the 3-D space filling model in (B). By convention, anterior daughters are placed on the left branches. Cell deaths are indicated with “X”.

With the expectation that detailed expression analysis might suggest possible targets for *mir-57*, we began our investigation of the gene by determining its embryonic expression pattern with cellular resolution at one-minute time intervals [16], [17]. Stably integrated lines with a 2.26 kb fragment upstream of the *mir-57* mature sequence fused with a fluorescent reporter mCherry [18] showed expression in the posterior cells of the embryo in a variety of tissues (Figure 1). Automated analysis of 3D time-lapse movies followed by manual editing revealed that the reporter was expressed in a bilaterally symmetric pattern in the posterior daughters of sublineages of AB and C founder cells, beginning at about the 200-cell stage (Figure 1F). The cells from these sublineages lie in the posterior part of the embryo only and represent a wide variety of cell types, including tail seam cells, the hypodermal cells hyp10 and hyp11, the cells producing the tail spike, rectal cells, the P11/12 cells and even body wall muscle cells (Figure 1F, Figure S1). Inspection of the movies beyond the comma stage also showed expression in the intestinal cells after elongation (data not shown).

Examination of larvae and adults showed that the reporter expression remained confined to the posterior of the animal with the exception of weak expression in more anterior intestinal cells (Figure 1E). Signal appears to increase through the L2 stage, after which it decreases with only minimal levels detectable in the tails of adults in hermaphrodites (data not shown). By contrast in males the adult tail shows high levels of expression (Figure S2).

To confirm that the expression patterns of the promoter-reporter fusion reflect those of the native *mir-57* expression, we performed *in situ* hybridization on whole mounted embryos using an LNA-modified probe. This method lacks the cellular resolution obtained through the automated lineaging system, but staining was clearly most pronounced in the posterior of the embryo in a pattern consistent with the results of the reporter assay (Figure 1C), while no apparent staining was observed in the *mir-57* deletion strain (Figure 1D).

### *mir-57* regulates posterior patterning

**Figure 2:**
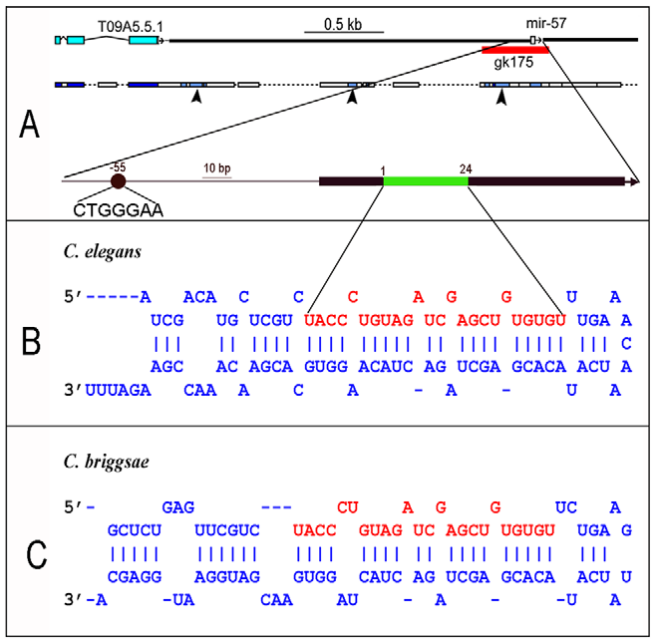
The *mir-57* locus and its predicted stem loop sequences. (A) *mir-57* locus. The 414 deletion, *gk175*, shown as a red bar, removes the entire structural gene. Regions of conservation with homologous regions of *C. briggsae* (WABA alignments) are indicated below (arrowheads). The region containing the *mir-57* gene is expanded below, showing the mature miRNA (green) within the predicted stem-loop region (black bar). A predicted LAG-1 binding site 55 bp upstream of the *mir-57* mature RNA is shown as a solid black circle. (B,C) predicted stem-loops for *C. elegans mir-57* (B) or its equivalent in *C. briggsae* (C). The mature *mir-57* sequences are highlighted in red. The predicted stem-loops are modified from MirBase. Note the *mir-57* mature sequences are identical between the two species.

**Figure 3:**
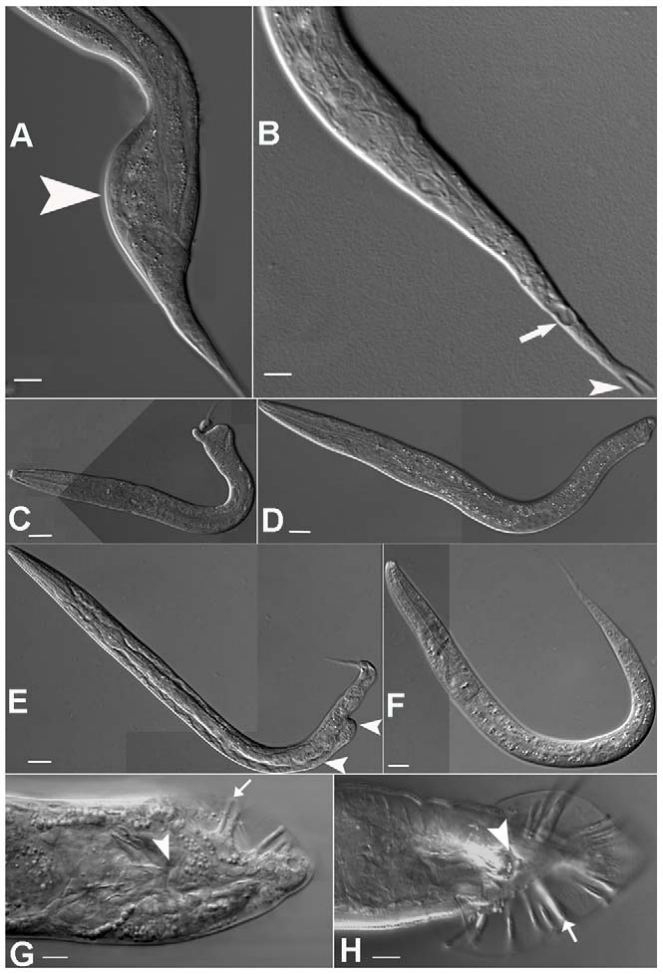
Phenotypes associated with *mir-57* loss of function and misexpression. Homozygous *mir-57(gk175)* animals placed at 26°C occasionally had preanal bulges (arrowhead in (A)) or vacuolated cells (arrow in (B)) in the tail. The animal in (B) also exhibited a forked tail spike (arrowhead). *mir-57* overexpression from extrachromosomal arrays resulted in Nob (C,D) and Vab-like (E) tail phenotypes as indicated by arrowheads. A wild-type larva (F) is shown for comparison. Males carrying the *mir-57* extrachromosomal arrays produced abnormal tails (G) compared to wild type (H). The anus is indicated by arrowheads and tail rays by arrows. Scale bars indicate 20 µm in all pictures.

To gain insight into the possible functional roles of *mir-57*, we examined animals homozygous for a presumptive null allele, *gk175*, a 414 bp deletion that removes the entire stem loop structure of the miRNA (Figure 2A). The mature sequences of *mir-57* are identical between *C. elegans* and *C. briggsae* (Figure 2B and 2C). In agreement with previous results [15], at lower temperatures (15° and 20°C) we observed no obvious phenotype associated with the homozygous mutant and similar or only slightly increased rates of embryonic or L1 larval arrest and adult sterility compared to wild type hermaphrodite animals (Table 1). However, at 26°C the mutants exhibited significantly increased rates of arrest and sterility (p<0.05, Student's T test). Many animals arrested as embryos before elongation (data not shown). Those arrested as larvae often had abnormal tails (23 of 47 examined). Most commonly, the tail contained a preanal bulge and vacuolated regions (Figure 3A and 3B). In about 5% of the arrested larvae, we observed a forked tail spike, something we have never observed in wild type (Figure 3B). Because we had also observed expression of *mir-57* in the adult male tail, we wondered if the male tail morphology might be a more sensitive assay for *mir-57* activity. However, of *mir-57* null males that develop into adulthood and were recognizably male, we found no defects in their tail structures compared to wild type at either room and elevated temperature (26°C, n = 38). In summary, the spatial correlation observed between *mir-57* expressing cells in embryos and the defects associated with its loss of function suggests a likely role of *mir-57* in regulating posterior cell fate specification in development.

**Table 1:** Characterization of *mir-57*(*gk175*) phenotypes.

Genotype	*mir-57(gk175)*			N2		
	Emb(%)	Lav(%)	Ste (%)	Emb(%)	Lav(%)	Ste (%)
15°C	1.6(514)	0.5(325)	2.7(339)	1.2(277)	0.8(363)	0(710)
20°C	1.3(840)	2.0(359)	5.3(472)	0.7(647)	0.5(451)	1.2(389)
26°C	6.7(892)	5.6(841)	32.1(978)	1.3(360)	1.5(399)	5.7(451)

Number of worms scored is indicated in parentheses.

Because the loss-of-function allele for *mir-57* only produced moderate defects in embryos and the larval tail, we reasoned that the effect of the allele is possibly masked by other miRNAs or factors that function redundantly with *mir-57*. An alternative approach to defining the roles of functionally redundant genes is to over express individual genes. Therefore, we examined the phenotypic effects of *mir-57* overexpression. In contrast to the low copy, integrated transgenes generated by bombardment that were used to assay expression, we used microinjection to create extrachromosomal arrays, which are expected to have many copies of the transgenes. Arrays containing the intact structural gene along with the upstream region (−2,260 to +234) produced pronounced defects exclusively in posterior region that resembled those seen in mutations of a posterior Hox gene, *nob-1*, which produces Vab (Variably ABnormal in tail) and Nob (NO-Back end) animals (Figure 3C–3F and see below). These phenotypes were typically more severe and affected a higher proportion of the adults at 20°C than were seen in the null allele at 26°C (Table 1 and Table 2). *mir-57(gk175*) animals injected with the same fragment also produced a comparable frequency of Vab progeny (Table 2). Adult male worms carrying the array often failed to develop proper tail rays (Figure 3G and 3H) and were unable to mate successfully to produce progeny (n = 34). The average ray number of each side of a male tail in the array containing animals is 3.2±1.2 (n = 38) compared to the 8.6±1.2 (n = 57) in wide type animals. The array containing males also produce only a few sperm (Figure S3A) compared to wild type (Figure S3B). Both the tail defects and the paucity of sperm likely contribute to the inability of *mir-57* overexpressing males to produce progeny. No defects were seen in the anterior body of either hermaphrodites (112) or males (n = 320) that carry the arrays, consistent with the expression patterns of *mir-57*.

**Table 2:** Phenotypes of *mir-57* transgenic arrays.

Promoter	miRNA	Position	Lines	Vab/Nob%	Background
*mir-57*	*mir-57*	−2260 to 234 b	8	29.3(625 d)	wild type
*mir-57*	none	−2260 to −63 b	5	33.9(373)	wild type
*mir-57*	*mir-57*	−2260 to 234 b	2	36.4(522)	*mir-57(gk175)*
*mir-57*	none	−2260 to −63 b	6	0(583)	*mir-57(gk175)*
*vab-7*	*mir-57*	−3664 to 36 c	3	11.3(362)	wild type
*vab-7*	*mir-57m* a	−3664 to 36 c	7	0(347)	wild type
*vab-7*	none	−3664 to 36 c	5	0(831)	wild type

**^a^**Mutated *mir-57* mature sequence (see Materials and Methods).

**^b^**Coordinates in base pairs relative to the start of the mature *mir-57* sequence.

**^c^**Coordinates in base pairs relative to the translational start of *vab-7* sequence. The sequences are fused with *mir-57* genomic region (−50 to 234 bp relative to the start of mature *mir-57* sequence).

**^d^**Number of animals scored.

**Figure 4:**
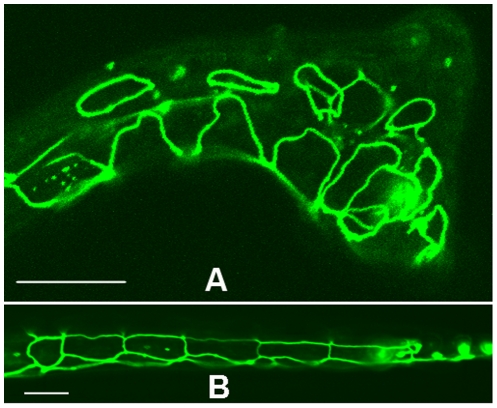
Hypodermal defects associated with *mir-57* overexpression. (A) A Nob animal produced by injection of the *mir-57* promoter segment shows disorganized hypodermal cells in the tail as labeled with *ajm-1*::GFP. (B) Uninjected control animal shows well organized hypodermal cells in the tail. Anterior is to the left. A 10 µm scale bar is indicated in each picture.

To examine the cell patterning defects in the tail, we injected the *mir-57* promoter (a 2260 bp fragment upstream from the miRNA mature sequence, see below) into a strain expressing hypodermal marker, *ajm-1*::GFP. As expected, the injection produced Vab and Nob animals as did in N2 animals (Table 2). The marker showed that the hypodermal cells are severely disorganized in the tails of Nob/Vab animals as opposed to those in uninjected control animals (Figure 4), supporting a role of *mir-57* in patterning posterior cells.

### An intact *mir-57* structural gene is required for producing Nob/Vab phenotypes

The tail defects associated with the *mir-57* arrays could derive either from elevated expression of the transgene or from the action of other sequences/elements in the 2.26 kb construct, which contains several regions conserved in *C. briggsae* (Figure 2). To test the first possibility, we drove *mir-57* expression by a *vab-7* promoter. The *vab-7* gene, an even-skipped homolog, is expressed in the posterior of the embryo in regions overlapping with those expressing *mir-57*, as determined by automated lineage-based expression analysis of a *vab-7* promoter::mCherry fusion integrated transgene (Figure S4, See Text S1). Extrachromosomal arrays of the p*vab-7*::*mir-57* construct produced Vab/Nob animals, albeit at a lower rate than the native *mir-57* promoter. The transgenic animals also yielded other phenotypes, including Dpy (12%, n = 262) and molting defects (3%, n = 340) (Figure S5). Importantly, a control construct with a mutated mature sequence of *mir-57* produced no Vab/Nob animals (n = 347, Table 2), indicating that presence of *mir-57* structural gene on the array is essential for the development of tail defects. Therefore, the tail defects can arise either from ectopic expression of *mir-*57 driven by *vab-7* promoter or its overexpression produced by the action of other sequences/elements in the 2.26 kb *mir-57* promoter. We speculate that the difference in the details of the phenotypes results from the differences in *vab-7* expression pattern from that of the *mir-57* promoter.

To test the effects of the putative *cis*-elements within the upstream region, we created constructs containing the upstream region separate from the structural gene. Surprisingly, injection of fragments (−2260 to −63) lacking the transcribed portion of *mir-57* and a predicted upstream LAG-1 binding site yielded a comparable fraction of animals with abnormal tails (Table 2). We postulated that the high copy number of the *mir-57* promoter fragment could have disrupted normal regulation of the genomic copy of *mir-57*, possibly titrating out an important inhibitory factor. Therefore, we introduced both the full length and truncated arrays into a background containing the genomic *mir-57* deletion allele, *gk175*. The full promoter-gene construct produced abnormal tails at rate similar to that seen in the wild type background. However, the absence of the genomic *mir-57* gene completely abolished the Vab and Nob phenotypes produced by the *mir-57* promoter fragment (Table 2), showing these effects required the presence of an intact copy of *mir-57* either in the genome or on the array. Thus, the abnormal tail phenotypes produced by the *mir-57* promoter fragment require at least one functioning copy of the *mir-57* structural gene.

### *mir-57* promoter arrays produced overexpression and ectopic expression of *mir-57*

**Figure 5:**
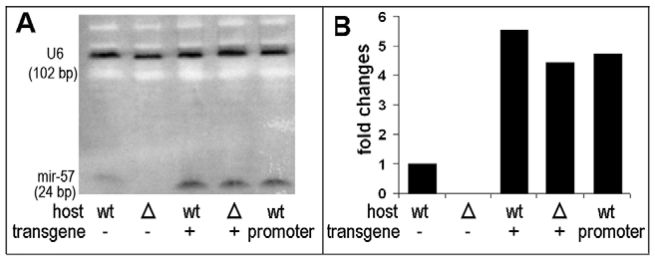
Northern blot results for *mir-57* overexpression. (A) 20 µg total RNA from wild type (wt) or *mir-57* mutant (Δ) L4 worms with (+) or without (-) injection of genomic DNA containing both regulatory and structural sequences of *mir-57* or with the injection of *mir-57* promoter only were separated on a 15% urea denaturing gel, blotted and hybridized using an antisense *mir-57* LNA probe and an antisense U6 DNA probe (See Materials and Methods). In order the lanes show wild type; the *mir-57* deletion strain; animals with transgenic arrays of the *mir-57* genomic fragment in the wild type background; animals with transgenic arrays of the with *mir-57* genomic fragment in the *mir-57* deletion background; and finally animals with arrays of the *mir-57 *promoter in a wild type background. Arrays of the *mir-57* genomic fragment into both wild type and the *mir-57* deletion mutant resulted in elevated levels of the *mir-57* miRNA. Arrays of *mir-57* promoter alone in the wild type background produced *mir-57* miRNA levels similar to that seen using the intact *mir-57* region. No signal was detected for *mir-57* deletion strain. (B) Quantification of expression changes measured by the intensities of the *mir-57* signal from lanes in panel A and normalized against U6 (See Materials and Methods).

To test more directly the hypothesis that the injected *mir-57* upstream sequence produces increased levels of *mir-57* miRNA, we performed Northern blot using a radioactive labeled LNA probe to detect the miRNA expression directly. As expected, both wild type and *mir-57* mutant animals injected with the full genomic region of *mir-57* (−2260 to +234) showed much higher levels of the miRNA transcripts, i.e., about 5 times higher than that of the un-injected controls. No signals were detected for the *mir-57* deletion strain (Figure 5). In addition, injection of the regulatory region alone (−2260 to −63) into the wild type animals also produced high levels of the miRNA transcripts, comparable to that seen for injections with the full *mir-57* region (Figure 5, Table 2). Given the partial transmission of the extrachromosomal arrays, the overexpression level for the array-containing animals is likely to be even higher than the increases measured for the populations of worms as a whole. These results strongly support the hypothesis that the array of the extra *cis*-regulatory fragments titrates out repressors controlling the endogenous expression of *mir-57*, thus increasing *mir-57* expression. Dissecting the molecular identities of the repressors is beyond the scope of this paper.

Next, to observe directly the effects of the *mir-57* extrachromosomal arrays on the activity of a genomic copy of the *mir-57* promoter, we introduced the *mir-57* promoter array into a background containing the integrated *mir-57*::mCherry reporter construct. Because of the instability of the promoter array, we did not carry out automated lineage-based expression analysis. However, examination of many individual expressing animals (n = 122) showed a fraction of animals (consistent with the inheritance of the array) with much earlier, more intense and more anterior expression of the reporter (Figure S6). Taken together these results show that overexpression of *mir-57* either from the arrays directly or from extra copies of the promoter region leading to the overexpression of the endogenous *mir-57* gene can perturb posterior development and thus produce Vab/Nob phenotypes.

### Interactions of *mir-57* with other factors involved in posterior patterning

Because these results implicate *mir-57* in posterior development, we examined its relationship to other genes with a known role in posterior embryonic development in *C. elegans*. These include genes in the *Notch* and *Wnt* pathways that provide signals that differentiate anterior daughters from posterior daughters as well as homeobox and Hox related genes involved in posterior patterning [19]–[24]. We used RNAi against genes of the *Notch* and *Wnt* pathways that specify cell lineage fates to look more broadly for interactions between these pathways and *mir-57*. RNAi against *pop-1*, a gene involved in *Wnt* signaling and required for anterior-posterior lineage fate polarity [19] converts ABp(l/r)ap lineages to ABp(l/r)pp lineages, as judged by both lineage fate and expression patterns (Figure S7). The RNAi is quite effective as evidenced by the complete homeotic lineage fate transformation from MS to E. These results indicate that the correct expression of *mir-57* is dependent on the lineage fate specified by the *Wnt* signaling pathway. Similarly, RNAi against *lag-1*, a gene involved in *Notch* signaling, converts ABplap and ABplpp to ABalap and ABarpp fates respectively with concomitant loss of *mir-57* expression in these altered lineages (Figure S7). In the C lineage, *mir-57* expression is dependent on the cell fates specified by PAL-1 as RNAi against the gene completely abolished the *mir-57* expression with concomitant cell fate changes as judged by the loss of asymmetry of cell cycle timing between Cxa and Cxp (Figure S8). Thus, in all these cases, *mir-57* expression is dependent on the lineage fates and thus likely downstream of these decisions.

These results suggest that *mir-57* might be a direct target of one or more of these early regulators. Computational analysis revealed the presence of a putative LAG-1 binding motif 55 bp upstream of the *mir-57* mature sequence (Figure 2A), suggesting that *lag-1* might directly regulate *mir-57* expression. To test the hypothesis, we produced a modified *mir-57*::HIS-24::mCherry fusion construct with the LAG-1 site removed by site-directed mutagenesis and used it to generate transgenic lines by microinjection. Arrays without the LAG-1 site also yielded high fractions of Vab/Nob progeny (data not shown), consistent with our earlier observations with the promoter region and indicating that this site is not responsible for the observed Vab/Nob phenotypes. However, arrays lacking the LAG-1 site failed to express the reporter in the tail, whereas the wild type promoter yielded strong expression, indicating that *mir-57* is likely a direct target of LAG-1.

### *nob-1* is co-expressed with *mir-57* spatially but with an earlier onset

**Figure 6:**
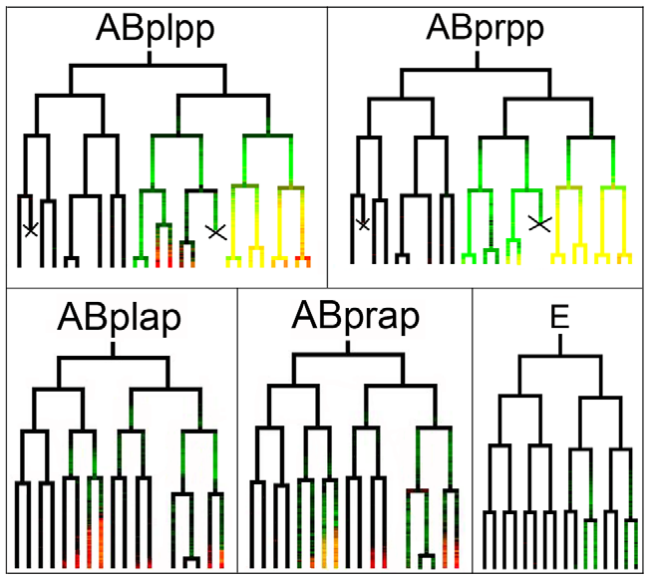
Embryonic lineage-based expression of *nob-1*. Shown are the superimposed lineage based expression trees between NOB-1::GFP (green) and *mir-57*::HIS-24:mCherry (red). The branch lengths (division timing) of *nob-1* and *mir-57* expressing trees are normalized against Sulston's lineage trees and the expression values are interpolated accordingly. In lineages expressing both genes, such as the ABpl/rpppp lineages, *nob-1* signal appears a full cell cycle before the onset of *mir-57* expression. Cell deaths are indicated with “X”.

In addition to the *Notch* and *Wnt* pathways, the *nob-1* gene, an ABd-B Hox gene homolog, is known to be involved in posterior pattern specification, with loss-of-function alleles resulting in embryonic lethality and larval arrest [22]. A hypomorphic allele, *ct230*, produces both Nob and Vab phenotypes quite similar to those that result from *mir-57* overexpression, suggesting that the *mir-57* and *nob-1* might function in the same pathways. To explore this possibility, we profiled the expression of *nob-1* with resolution comparable to that of *mir-57* by automated lineage-based expression analysis using an integrated NOB-1::GFP protein fusion transgene (see Materials and Methods). The reporter was detectable from roughly the 100-cell stage in posterior progeny of ABp(l/r)pp and ABp(l/r)ap, the same sublineages in which *mir-57* is also expressed (Figure 6). These results confirmed and refined the *nob-1* expression pattern determined independently using a similar rescuing NOB-1::GFP construct (E. Kress, L. Edgar and W. B. Wood unpublished). Despite the striking overlap of expression between *mir-57* and *nob-1* in the AB sublineages, their temporal expression patterns are quite different. The *nob-1* reporter appears at about the 100-cell stage with a fairly uniform time of onset in different cells whereas *mir-57* expression mostly appears after the 200-cell stage, although the more posterior the cells are, the earlier onset is detected. Expression of *nob-1* was either very weak or not observed in the C lineage but was seen in the posterior E lineage by the 100-cell stage (Figure 6). As described above, expression of *mir-57* was clearly present in the C lineage and only expressed in the E lineage after elongation (data not shown) so that spatial expression of *nob-1* and *mir-57* are not totally overlapping in these lineages

### *nob-1* RNAi downregulates *mir-57* expression

**Figure 7:**
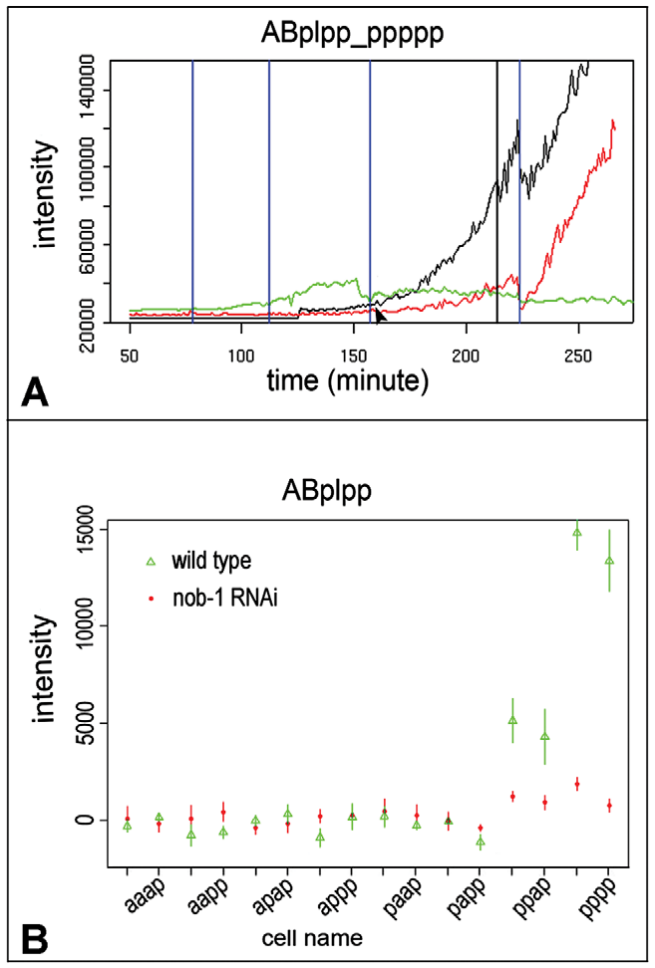
Effect of *nob-1* RNAi on the expression of *mir-57*. (A) expression intensity of *mir-57* (black) and *nob-1*(green) in ABplppppppp and its ancestors is plotted against developmental time in minutes at 20°C. The altered expression of *mir-57* after RNAi against *nob-1* is similarly plotted (red). Vertical blue lines mark cell divisions. The vertical black line indicates the time point for the measurement in B (below). (B) *mir-57* expression in all the progeny of ABplpp at the indicated time point (215, the vertical black line in (A)) between the RNAi treated (red) and untreated animals (green). For simplicity, cell names include only the extension from ABplpp. The expression intensities were derived from the average values of six RNAi treated and six untreated image series and standard deviations are shown as vertical bars (See Materials and Methods).

Given their similar expression patterns in the AB sublineages and the earlier onset of *nob-1* expression compared to that of *mir-57*, we reasoned that *nob-1* might be required for *mir-57* expression. To test this hypothesis, we depleted *nob-1* activity by RNAi and observed its effects on the *mir-57* reporter expression with time using automated lineaging. The RNAi produced Vab/Nob progeny at rates comparable to the *ct230* hypomorphic allele (data not shown). The AB sublineage division patterns resembled those of wild type but the *mir-57* reporter expression onset was delayed and expression level in the AB sublineages was significantly reduced (n = 6, p<0.01, Student's t test) compared to untreated animals (Figure 7), suggesting that *mir-57* activity is at least partially dependent on *nob-1* activity in these lineages. The residual expression of *mir-57* might reflect the incomplete penetrance of the RNAi. Alternatively other factors may be responsible for the remaining activation. RNAi against *nob-1* did not affect *mir-57* expression in the C lineage (data not shown). Other factors such as PAL-1 may be involved in the control of *mir-57* expression in this sublineage (Figure S4 and Figure S8).

### *nob-1* is a direct target of *mir-57*

**Figure 8:**
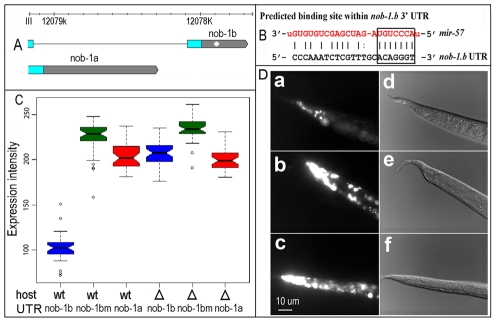
Validation of *nob-1* as a target of *mir-57*. (A) Two alternative forms of *nob-1* 3′ UTRs (grey bars) are shown in scale. Blue bars are coding exons and black lines introns. A putative *mir-57* binding site within the *nob-1b* UTR is shown as a white star. (B) Sequence of a putative *mir-57* binding site within the *nob-1b* 3′ UTR (chrIII:12077862-12077884, WS205). The mature *­mir-57* sequence is highlighted in red. Putative seed sequences are enclosed in the rectangle. (C) Effect of the binding site on the reporter expression. Shown are boxplots of tail expression intensities derived from three different 3′ UTRs: *nob-1*b, *nob-1*bm (*nob-1*b UTR with site-directed removal of the binding site), *nob-1*a injected into wild type (wt, first three columns) or *mir-57* mutant (Δ last three columns) animals as indicated (See Materials and Methods). (D) Micrographs of reporter expression in wild type (a and b) or *mir-57* mutant animals (c). Animals were injected with constructs carrying wild type (a) or mutated *nob-1*b UTR (b). The same array as that used in (a) was crossed into *mir-57* mutant animals (c). d, e, f are DIC pictures of a, b and c respectively. Vab tails are caused by *mir-57* promoter array.

Given that miRNAs generally act by reducing gene expression and that overexpression of *mir-57* produced tail defects similar to those of *nob-1* reduction-of-function mutations, we reasoned that overexpression of *mir-57* might inhibit *nob-1* expression, and thus produce the observed Nob/Vab phenotypes. Consistent with this, the *nob-1b* 3′ UTR, one of the two alternative *nob-1* 3′ UTRs, contains a predicted *mir-57* binding site [7] (Figure 8A and 8B). The putative binding site for *mir-57* has the highest score among all the predicted miRNA binding sites within the *nob-1* 3′ UTR [7]. To explore experimentally the functional role of the *mir-57* binding site, we used *mir-57* promoter driven reporter constructs followed by *nob-1* 3′ UTRs to induce strong overexpression of the endogenous *mir-57* in order to produce a robust effect. We made three constructs: two of them using the 3′ UTRs from the different splice isoforms, *nob-1a* and *nob-1b* and a third construct using the 3′ UTR from *nob-1b* in which the candidate *mir-57* binding site was removed by site-directed mutagenesis (See Materials and Methods). These constructs were introduced into the wild type N2 background by microinjection to create extrachromosomal arrays, which were then crossed into the *mir-57* deletion background. In the wild type background, the arrays, because they contain the *mir-57* promoter region, should result in overexpression of *mir-57* from the genomic copy. As predicted, expression of the reporter gene is significantly reduced by the presence of the binding site in *nob-1b* (p<0.01, Student's two-tailed t test, Figure 8C and 8D). The reduction in reporter construct expression was not observed in the absence of a functional *mir-57* gene, indicating that *nob-1* is a direct functional target of *mir-57*. A caveat for these experiments, however, is that all DNA constructs contained a *mir-57* promoter introduced by microinjection, which as we described above leads to overexpression of *mir-57*. Thus, these results may exaggerate the effects of a more physiological level of *mir-57* expression, but the results clearly show that the binding site is functional *in vivo*.

We looked genetically for further support that *nob-1* is a target of *mir-57*. By this hypothesis, *mir-57* loss of function mutations might lead to increased expression of *nob-1* although in contrast to the overexpression experiments, such an effect might be attenuated by other, redundant regulators of *nob-1*. In agreement with this, overexpression of *nob-1* from an extrachrormosomal array partially mimics the phenotypes of *mir-57* loss of function albeit at a lower level (Table S1, n = 3). However, we did not observe male tail defects in *nob-1* overexpressing strains, suggesting that *mir-57* may regulate male tail development independent of *nob-1*. The lower penetrance might reflect partial transmission of extrachromosomal array, alternatively, other targets of *mir-57* may also contribute to the observed phenotypes. Further, using a reporter to assay *nob-1* expression, the loss of function mutation in *mir-57* only produced modest effects on the *nob-1* expression (Figure S9, See Text S1), suggesting the miRNA functions redundantly with other miRNAs or pathways.

**Figure 9:**
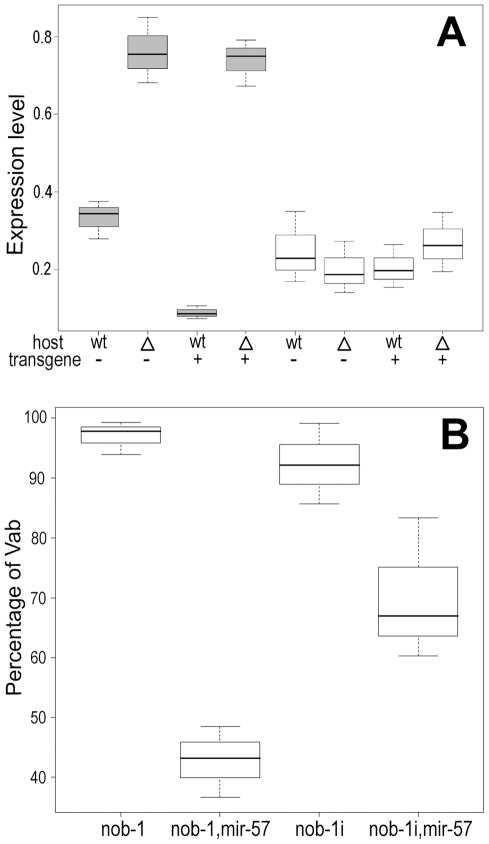
Genetic interactions between *nob-1* and *mir-57*. (A) Real time RT-PCR assay of *nob-1a* (white) and *nob-1b* (grey) transcripts in the presence (wt) or absence (Δ) of genomic *mir-57*. Deletion of genomic *mir-57* increases *nob-1b* transcripts about two-fold while no effects were observed for *nob-1a* transcript level. The *nob-1b* transcript level is roughly four-fold lower in *mir-57* promoter-array wild type strain (+) than that in wild type only (−). (B) The percentage of Vab animals is plotted for mutants or RNAi of *nob-1* in the presence or absence of genomic *mir-57*. The left two of experiments contrast the phenotypes of *nob-1(ct230)* and *nob-1*(*ct230*); *mir-57*(*gk175*), whereas the right two contrast *nob-1*(RNAi); and *nob-1*(RNAi); *mir-57*(*gk175*). The fraction of *nob-1* mutant animals exhibiting abnormal tail phenotypes is reduced in both cases by the removal of the genomic *mir-57*.

To look for biochemical evidence that *nob-1* is a target of *mir-57*, we measured the endogenous levels of the two alternative *nob-1* transcripts, *nob-1a* and *nob-1b* in the presence or absence of the genomic *mir-57* copy using real-time PCR assay. Deletion of *mir-57* significantly increases the *nob-1b* transcript level (p<0.01, Student's t test, Figure 9A) and *mir-57* promoter arrays in the N2 background significantly decrease its transcript level, roughly four fold (p<0.05, Student's t test, Figure 9A). As expected, deletion of *mir-57* had no effect on the *nob-1a* transcript, which does not contain a *mir-57* binding site.

As a potentially more sensitive test of the interaction at more physiological levels, we constructed doubly mutant *mir-57(gk175)*; *nob-1(ct230)* animals, postulating that if *mir-57* normally is responsible for down regulation of *nob-1* activity, removal of *mir-57* activity might ameliorate the effects of the hypomorphic allele. We found that the doubly mutant animals showed a significant reduction in the fraction of progeny exhibiting the Nob phenotypes compared to *nob-1(ct230)* animals (p<0.01, Student's t-test) (Figure 9B). Similarly, the penetrance of *nob-1* RNAi induced phenotypes were reduced in the *mir-57(gk175)* background compared to the wild type animals. One explanation of this suppression of the hypomorphic *nob-1* mutant is that the deletion of *mir-57* prevents repression of *nob-1* expression, partially restoring *nob-1* activity in the mutant. Perhaps the *nob-1* hypomorphic allele creates a sensitized background, exaggerating the impact of *mir-57* loss of function, even in the presence of other factors redundantly regulating *nob-1* activities.

## Discussion

The detailed analysis of the expression pattern of *mir-57* and *nob-1*, coupled with the phenotypes observed in the absence and overexpression of *mir-57* demonstrate that the miRNA gene plays a role in combination with the posterior Hox gene to specify posterior identity. The expression patterns reported here generally agree with previously described patterns [25] but with much higher temporal and spatial resolution, allowing us to infer and test the detailed functional relationships with other genes operating in related pathways. For example, both *mir-57* and *nob-1* are expressed in the same AB sublineages, ABpl(r)ap and ABpl(r)pp, which give rise to a variety of tissue/organ types in the posterior region, the region where the abnormal phenotypes were observed. Based on the detailed expression map as well as their similar phenotypes, we examined the interaction between *mir-57* and other genes implicated in posterior development in *C. elegans* including *­nob-1*. The expression of *mir-57* in the posterior sublineages of AB founder cells was dependent upon their proper specification through the *Notch* and *Wnt* pathways. In addition the expression of the *mir-57* transgene required the presence of a binding site within its promoter sequence for the *Notch* pathway factor LAG-1, suggesting that the gene might be directly regulated by the pathway. More dramatically, reduced function of *nob-1* delayed the onset and reduced the level of *mir-57* expression and in turn, *nob-1* is to be a direct target of and repressed by *mir-57*. This would constitute a negative regulatory loop between the miRNA and the Hox gene in providing positional cues for posterior development. The results suggest that the Nob/Vab phenotypes produced by overexpression of *mir-57* are likely caused at least in part by down regulation of *nob-1* activities within the posterior region, mimicking those of hypomorphic or null *nob-1* mutants. This is supported by the evidence that *mir-57* promoter injection produced a significant decrease of *nob-1b*, but not *nob-1a*, transcripts (Figure 9A). Similarly, the Emb phenotype observed for the *mir-*57 null mutant at 26°C could be partially reproduced by *nob-1* overexpression. Further support for *mir-57* regulation of *nob-1* comes from the fact that the *mir-57* mutation partially alleviated the phenotypes of *nob-1(ct230)*, a hypomorphic allele, presumably resulting from partial release of the repression of *nob-1*. Thus, in normal development, our data suggests that *nob-1* expression begins by the 100-cell stage, activates *mir-57* by the 200-cell stage, which in turn dampens the expression of *nob-1*, perhaps in conjunction with other miRNAs. This down regulation might function to reinforce the transcriptional silencing of *nob-1* activity in late embryonic stages when it may no longer be required to provide positional cues for posterior fate specifications. Such reciprocal regulation between the Hox gene and the miRNA might provide more robust control over the regional identity than the Hox gene alone. It should be noted that *mir-57* expression is very likely subject to control of positional cues other than *nob-1* because expression onset of *nob-1* seems synchronized but that for *mir-57* are not (Figure 6).

In vertebrates, miRNA genes have also been found to have a role in Hox gene regulation, helping to reinforce posterior prevalence [26]. Notably, the closest homolog to *mir-57* in other species is the broadly conserved gene miR-10^7^. miR-10 has been found to repress Hox gene activities in zebrafish spinal cord[27]. In mouse, miR-10a and another miRNA gene, miR-196a, are embedded in a Hox gene cluster and their expression is apparently overlapping with those of Hox genes [28]. In addition, miR-196a represses Hoxb8, indicating its restricted expression pattern likely reflects a role of microRNA in the patterning of the Hox complex. Interestingly, miR-126 has been shown to regulate Hoxa9 by binding to its target sites within the homeobox domain [29]. A negative regulatory loop between miRNA genes and other transcription factors has also been described in vertebrates [30], [31]. By miRNA profiling using an E2F1-indicible Saos-1 cell line, miRNAs *miR-449a/b* were identified as direct transcriptional targets of E2F1 [30]. *miR-449a/b* negatively regulates E2F activity through a feedback loop mechanism by targeting oncogenic CDK6 and CDC25A, leading to dephosphorylation of pRb, which is required for activation of E2F-responsive genes to promote cell cycle progression. miR-133b is also involved in a feedback regulatory loop that includes a paired-like homeodomain transcription factor Pitx3 in mammalian midbrain dopaminergic neurons [31]. Thus, such a negative feedback regulation between miRNAs and homeodomain transcription factors may provide a conserved mechanism to control metazoan position specific patterning. Although *mir-57* is not found within a Hox gene cluster, Hox genes in *C. elegans* are only relatively loosely clustered [32].

Like many miRNA genes, *mir-57* most likely functions redundantly with other miRNA genes. Deletion of the gene had little effect on the worm's fitness except at high temperatures and even here the defects were minor and penetrance incomplete. Overexpression of the gene produced more dramatic effects, but the overexpression phenotypes of miRNAs must be interpreted with caution since high miRNA levels may create off-target effects. However, the observed phenotypes were consistent with the expression pattern. A more systematic investigation of the high resolution expression patterns of the full set of miRNA genes would provide valuable insight into the likely partners, just as the detailed expression information here provided hints as to function.

The ability of the 2.26 kb sequence upstream of the mature *mir-57* miRNA to produce Vab/Nob phenotypes is intriguing. Although no evidence of another gene in this region has been found in extensive RNA-seq studies[33], we cannot entirely rule out the presence of such a gene. Nonetheless, the dependence of the resultant phenotypes on the presence of a functional copy of the *mir-57* gene, either in *cis* or in the genome, argues that the presence of the fragment in high copy number results in *mir-57* misexpression. Our Northern blot results provide direct evidence that injection of either the *mir-57* genomic region including its mature sequence and flanking sequences or the promoter region of *mir-57* alone produced overexpression of the miRNA, which likely underlies the tail defects associated with these assays. The ability of the p*vab-7*::*mir-57* arrays to partially mimic the phenotypes also implies that overexpression of *mir-57* is the primary cause for the observed defects. This would be consistent with the 2.2 kb fragment binding and titrating out a repressor of *mir-57* but what that factor might be remains unknown.

Although the *nob-1* gene seems to be one direct target of *mir-57*, there are undoubtedly many other direct or indirect ones both within the AB sublineages where both *mir-57* and *nob-1* are expressed and also in the C lineage where *mir-57* is found in the absence of *nob-1.* MirBase lists a total of 492 candidate targets for *mir-57* [7], [34] but which of these might be functional targets in the C lineage is unclear. Again detailed expression patterns would greatly restrict the list of possibilities. Taken together, by using the automatic high-resolution gene expression technology, we were able to identify a negative regulatory loop between *mir-57* and a Hox gene to control regional identity.

## Materials and Methods

### Strains and genetics

All the strains were maintained on NGM plates with OP50 *E. coli* at room temperature except for temperature sensitive assay of *mir-57* mutant phenotypes at the indicated temperature. The following strains were used in the assay: N2; VC347, *mir-57*(*gk175*) II; RW10029, *unc-119*(*ed3*), *stIs1007*[his-72::GFP, *unc-119(+)*], *stIs*10025[pie-1::GFP::his-58, *unc-119(+)*]; RW20050, *mir-57*(*gk175*) II; BW1379, *nob-1*(ct230) III; RW20051, *mir-57*(*gk175*) II, *nob-1*(ct230) III; RW10044, *unc-119*(ed3), *stIs10044*[p*mir-57*::HIS-24::mCherry, *unc-119(+)*]; RW10226, *unc-119*(ed3), *stIs10226*[HIS-72::mCherry, *unc-119(+)*]; BW2020, *ctIs57*[NOB-1::GFP, *rol-6* dm]; RW10044, *unc-119*(ed3), *stIs10044*[p*mir-57*::HIS-24::mCherry, *unc-119(+)*], *stIs10026*[HIS-72::GFP]; RW10174, *unc-119*(ed3), *stIs10174*[p*pal-1*::HIS-24::mCherry, *unc-119(+)*]; RW10199, *unc-119*(ed3), *stIs10199*[p*vab-7*::HIS-24::mCherry, *unc-119(+)*]; RW10226, *unc-119*(ed3), *ctIs57*[NOB-1::GFP, *rol-6* d], *stIs10226*[HIS-72::mCherry]; PS4657, *unc-119*(ed3), syIs78[ajm-1::GFP + *unc-119(+)*]. The ­*mir-57* (*gk175*) allele was backcrossed to N2 Bristol strain five times before phenotypic assay. The presence of ­*mir-57*(*gk175*) allele was followed by single worm PCR. The following phenotypes were scored at 15°C, 20°C and 26°C respectively for N2 and RW20050 strains: Emb (embryonic lethality), Lva (larva arrest), Ste (sterility).

### Transgenic constructs

A 2260 bp (−2260 to −1) fragment upstream of *mir-57* was cloned into the restriction sites upstream of a HIS-24::mCherry cassette using AvrII and SmaI sites in the pJM20 vector [17] to give rise to pZZ1. mCherry is a worm optimized derivative of Cherry [18]. Histone HIS-24 was used to direct the reporter signal into nucleus. pJM20 also contains transgenic selection marker *unc-119*(+). The pZZ1 (*Pmir-57*::HIS-24::mCherry, unc-119+) construct was bombarded into *unc-119*(*ed3*) worms to generate integrated reporter expressing strains for expression profiling. A total of 12 independent lines were generated and all of them showed the similar expression patterns. A single line was backcrossed three times with N2 and the resultant reporter expressing strain was mated into the lineaging strain, RW10029 [16], which ubiquitously expresses nuclear localized GFP to generate a strain RW10048 that are homozygous for both GFP and RFP loci. Similar method was used to generate reporter-expressing strains using RW10174 and RW10199 for automatic profiling of *pal-1*and *vab-7* expression respectively (See Text S1 for the primer used). To build a translational GFP fusion reporter strain for *nob-1*, an approximately 15 kb fragment spanning the entire coding region plus its upstream regulatory sequences was fused in frame with GFP followed by *unc-54* 3′ UTR. The construct was co-injected into N2 with pRF4(*rol-6* d(su1006)). The resulting transgenic strain was integrated into the genome by gamma irradiation to give rise to BW2020.

The constructs for site-directed removal of the LAG binding site and the mutated *mir-57* as well as for the hybrid construct between *mir-57* and *vab-7* promoter, were built by the fusion PCR technique as described below [35] (See Text S1 for the primers used). To build the construct with site directed removal of LAG-1 site within the *mir-57* promoter, the fragments flanking LAG-1 site were PCR amplified from pZZ1 and fused together by PCR. The resulting construct contains the full *mir-57* promoter (-2260 to -1) except for the LAG-1 site (See Text S1 for the primer sequences). To build mutagenized *mir-57* driven by *vab-7* promoter, two pairs of primers were designed so that they overlap on the mature *mir-57* sequence. The *mir-57* mature sequence was mutated into an unrelated sequence (See Text S1 for the primers used). To build the hybrid construct between *mir-57* and *vab-7* promoter, the 3400 bp *vab-7* promoter was fused with 284 bp *mir-57* stem-loop plus its flanking sequences. The *mir-57* overexpression construct is a 2494 bp PCR product that includes 2260 bp promoter sequences and the 234 bp stem-loop and its downstream sequence. The *mir-57* promoter only construct is the PCR product that is -2260 to -63 bp from the *mir-57* mature sequence. These PCR products were co-injected with pRF4(*rol-6* d) with concentrations of 20 and 100 ng/µl, respectively. The *mir-57* promoter only PCR product (−2260 to −63) was also injected into both wild type and *mir-57* deletion animals in the same concentrations. The overexpression phenotypes for hermaphrodite were scored from three independent transgenic lines synchronized at L2 stage.

### Embryonic expression profiling

Embryonic lineaging analysis and expression profiling of *mir-57* was performed with strain RW10048 using StarryNite and AceTree as described [17], [36] with modifications. Strain RW10048 was used for profiling of *mir-57* expression. Given the relatively late stage of *mir-57* expression during embryogenesis, we traced the relevant embryonic lineage until the last round of cell division in both “forward” and “backward” directions. In the “forward” direction, we traced the lineage using the standard method for those up to 350-cell stage. For the “backward” direction, we started with the reporter expressing cells at a late stage of embryogenesis, for example, comma stage, and traced the cells backward until the progenitors could be reliably linked to the nuclei identified by the forward tracking method. In this way we captured the expressing lineages at the late period of embryogenesis up to comma stage. To profile *nob-1* expression, we made a strain RW10226 that ubiquitously expresses histone::mCherry in somatic nuclei and crossed it into BW2020, a NOB-1::GFP expressing strain for lineaging using mCherry labeled nuclei. Due to the lack of the germline expression of mCherry, cell lineage from one to about 70 celled embryo were manually traced using DIC images. After the embryonic cell lineage was produced for both *mir-57* and *nob-1* expressing strains, pixel densities from GFP (*nob-1*) or RFP (*mir-57*) channel were extracted and aligned against the lineage tree branch for each time point and all nuclei. To compare the expression from different experiments, division timing was normalized against those derived from Sulston lineage tree [37] and expression values interpolated accordingly. A total of six and four embryos were profiled for *mir-57* and *nob-1* expressing embryos respectively. To assign the expression values of reporter expressing cells, we did the background subtraction (blot) to eliminate marginally expressing cells and thus increase the specificity of calling the expressing cells [17]. To profile the *mir-57* expression after the RNAi against *nob-1*, the embryos were mounted 24 hours after their parents were injected and imaged for six hours at room temperature. A total of six RNAi treated embryos were profiled for expression up to 450-cell stage for the selected sublineages. To plot expression of ABplpp_ppppp with time, we used both raw and background subtracted expression values and the two data sets agree well with one another in terms of relative intensity (data not shown).

### In situ hybridization

In situ hybridization was performed as described [38], [39] with following modifications. DIG labeled LNA-modified probe complementary to the mature *mir-57* was made by Exiqon with the sequence ACACACAGCTCGATCTACAGGGTA. The mix-staged embryos were immobilized on poly-lysine coated slides followed by methanol fixation. The hybridization and wash were performed at 37°C (20°C below the probe melting temperature) as suggested elsewhere [39].

### Northern blot

Worm populations synchronized at the L4 stage were incubated with acid phenol (pH 4.5) at 65°C for one hour followed by centrifugation. The top layer was transferred to Phase Lock GelHeavy tubes (Sigma-Aldrich) and centrifuged for 1 minute. The aqueous layers were re-extracted with phenol and chloroform followed by precipitation and washing. A total of 20 µg total RNA was loaded onto 15% polyacrylamide denaturing (urea) gel for each sample and transferred to Nylon (+) membrane by electroblotting in 1 X TBE buffer. The *mir-57* antisense LNA probe and U-6 antisense probe (GTCATCCTTGCGCAGGGGCCATGCTAATCTTCTCTGTATTGTTCCAAT) were 5′ labeled with γ-^32^P ATP. The two probes were mixed and hybridized to the membranes at 50°C overnight in the hybridization buffer (5 X Denhardt's, 2 X SSC, 0.1% SDS) and washed twice at 50°C in the wash solutions (2 X SSC, 0.1% SDS). The blots were visualized and signals quantified using Amersham Biosciences phosphorimager according to the manufacturer's instructions.

### RNAi

All RNAi experiments were done by microinjection as described [40]. The primers used for PCR amplification of genomic fragments for *nob-1, pal-1, pop-1 and lag-1* were derived from Ahringer's oligonucleotides [41] flanked with a T3 or T7 promoter at each end. The single-stranded transcript resulted from T3 and T7 RNA polymerase were pooled and annealed at 68°C for 10 minutes and 37°C for 30 minutes. The concentration for the injected dsRNA is 200 ng/µl in ddH_2_O. Two- or four- celled embryos were retrieved from adult worms after 16–24 hours of after injection by cutting the uterus and mounted for imaging for about six-hours.

### Characterization of male tails

The number of the tail rays was counted only on one side of the male tails from both N2 (n = 57), *mir-57*(*gk175*) (n = 32) and the *mir-57* promoter array containing animals (n = 38). For *mir-57* animals, the ray number was counted on both room temperature and 26°C. To test the fertility of the array containing males, 10 males were put in the same plate with 10 N2 hermaphrodite animals in 3 replicates and the presence of the roller or male animals were examined for the successful crossing. The fertility of the *mir-57* males was examined in a similar fashion but only the presence of male progeny was examined.

### Microscopy and imaging

All of the post-embryonic images were taken with a ZEISS Axioplan 2 compound microscope equipped with AxioCamHR camera using a 63X objective lens. The florescent images for embryos were taken with a ZEISS LSM510 confocal microscope. Four-D imaging for lineage analysis and gene expression profiling was conducted essentially as described previously [17].

### Quantification of *mir-57* expression

To test the effect of the predicted *mir-57* target site within the *nob-1* 3′ UTR on the reporter expression, the pZZ1 construct was modified so that the *let-858* 3′ UTR was replaced with the 3′ UTR either from *nob-1a* or *nob-1b* by the following methods. The fragment containing P*mir-57*::HIS-24::mCherry was PCR amplified from pZZ1. The 3′ UTRs from *nob-1a* or *nob-1b*, termed as *nob-1*a or *nob-1*b respectively were amplified from N2 genomic DNA (See Text S1 for the primers used). The UTRs were fused with the above reporter at the 3′ end by fusion PCR. The resulting fusion products were cut with BamHI and ApaI and ligated with pZZ1 cut with the same restriction enzymes to give rise to pZZ43 (nob-1a UTR) and pZZ44 (nob-1b UTR) respectively. Sited directed removal of a putative *mir-57* binding site within the *nob-1b* 3′ UTR was performed in the similar way as that used for LAG-1 site-directed mutation described above and was termed as *nob-1* bm hereafter. The three UTR constructs for *nob-1*a, *nob-1*b and *nob-1*bm were co-injected with pRF4 (*rol-6d*) into wild type (N2) with concentrations 20 (UTR constructs) and 100 (pRF4) µg/ul respectively. Three independent lines were obtained for each injection. The extrachromosomal arrays of the transgenic animals were crossed into the mutant *mir-57*(*gk175*) background and genotyped by single worm PCR. The three independent transgenic lines from each injection and crossing were individually synchronized by bleaching and eggs were put on unseeded plates overnight at room temperature. The hatched worms were transferred to seeded NGM plates and allowed to grow for 24 hours. The microphotographs for tail expression were taken using AxioCamHR camera using a 63X object with 919 ms exposure for each independent lines. A total of 39 animals from each independent line were used for taking micrographic photos. The RFP intensities in tail regions (measure from anus to tail end, if anus not available, measured from the end of intestine to the end of tail) were quantified using ImageJ and the data were averaged for box plotting with R package.

### Quantification of *nob-1* transcript

To quantify the change of *nob-1* transcript in the presence and absence of ­genomic *mir-57*, we performed Real-time RT-PCR using LightCycler (Roche) with QuantiTect SYBR Green Kit (Cat # 204143) and QuantiTect Reverse Transcription Kit (Cat # 205311). Four strains were used for total RNA extraction using RNeasy Fibrous Tissue Mini Kit (Cat # 74704): N2, VC347(mir-57(−/−)), as well as the above two strains injected with *mir-57* promoter. Worms were staged at L4 before total RNA preparation. For promoter injected strains, a total of 763 and 824 L4 array containing worms were picked for RNA preparations. The Real-time RT-PCR were performed in three triplicates using primer pairs specific for *nob-1a, nob-1b,* and *gpd-1* (a GAPDH encoding gene) based on the manufacture's instructions. In the case of array containing animals, the Real-time RT-PCRs were performed in triplicate using the same template. The primers sequences are gpd-1-L: TGTCGACTGATTTCGTGTCC; gpd-1-R: TCGACAACACGGTTCGAGTA; nob-1a-L: AGGCAGTATTCAGCGGAAAG; nob-1a-R: tgaaaatccagagaagctcaaa; nob-1b-L: TGCACAATTGATGCTTGATG; nob-1b-R: GTCGTTGACGCAGTTTCTTG. Expression levels are normalized against gpd-1 before statistical analysis.

### Genetic interaction between *nob-1* and *mir-57*

*nob-1(ct230)* and *mir-57(gk175)* double mutant was made by crossing and genotyped by single worm PCR. RNAi by injection against *nob-1* was performed as described previously for N2 and *mir-57* mutant animals. Vab or Nob (data not shown) phenotypes were scored for *nob-1(ct230), nob-1(ct230)* and *mir-57(gk175)* double, *nob-1(RNAi), nob-1(RNAi)* and *mir-57(gk175)* double animals. 10 young adults were plated on a single plate for each genotypes and allowed to lay eggs for 6 hours and picked off. The phenotypes were scored for three successive days at room temperature. Each experiment was performed in three replicates.

## Supporting Information

Figure S1 *mir-57* shows expression in a variety of cell types in the posterior sublineages. The figure is complementary to the Figure 1 but indicates the terminal cell fates of the expressing sublineages. Vertical bars denote scaled red intensity represented as arbitrary Boyle Unit.
(7.26 MB TIF)

Figure S2 *mir-57* expression in adult male tail. (A) DIC; (B) RFP; (C) merged.
(2.00 MB TIF)

Figure S3 Adult male phenotypes associated with *mir-57* overexpression. Note, *mir-57* overexpressing animals develop few tail rays and produced only a few sperms (B) compared to N2 (A) animals as indicated by arrow head.
(1.54 MB TIF)

Figure S4 Lineage expression of mCherry reporter driven by *vab-7* and *pal-1* promoter. (A) *vab-7* showed expression in C lineage except for the anterior half of hypodermal sublineages, i.e., Caaaa and Cpaaa. (B) *pal-1* showed expression in all P2 sublineage except for germline precursor Z2 and Z4 (not traced as far as other sublineages). Note that all of the reporter-expressing cells are located within the posterior part of embryo (data not shown).
(1.74 MB TIF)

Figure S5 Other posterior defects associated with injection of fusion between *vab-7* promoter and *mir-57* stem loop sequences. Shown are molting defects (A) and other tail abnormalities (B-D) as well as Dpy (E).
(4.30 MB TIF)

Figure S6 Injection of *mir-57* promoters caused earlier onset and ectopic expression (more anterior) of *mir-57* in the embryo. An approximately 350 celled embryo was photographed for *mir-57* expression in both wild type background animals (A–C) and those carrying the *mir-57* promoter array (D–F). The array containing animals were verified by following their tail phenotypes after hatching.
(4.20 MB TIF)

Figure S7 *mir-57* expression is dependent on the lineage fate. RNAi against *pop-1* produced homeotic lineage fate transformation, i.e., from ABplap into ABplpp (A,F) and MS into E like lineage (C, data not shown) (the later transformation serves as a reference for the RNAi effectiveness) while *mir-57* expression and lineage fate remained unchanged in ABplpp (B,G). Note: *mir-57* expression in ABplap becomes characteristic of that of ABplpp (A,F,G). RNAi against lag-1 transformed the posterior lineage fates of ABplap and ABplpp into those of anterior ones, i.e., ABalap (D) and ABarpp (E) respectively. *mir-57* expression is abolished in both lineages.
(1.59 MB TIF)

Figure S8 RNAi against *pal-1* altered the *mir-57* expression in C lineage. Compared to wild type (A), the treatment abolished the fate asymmetry between C derived hypodermis and body wall muscle as well as *mir-57* expression (B).
(3.77 MB TIF)

Figure S9 Effects of *mir-57* loss of function on the NOB-1 expression. Shown are the notched boxplots of NOB-1 expression in the presence and absence of *mir-57*. NOB-1 expression in N2 background is shown as green and the expression in *mir-57* deletion background (VC347) as red.
(0.81 MB TIF)

Table S1 Phenotypes of NOB-1 overexpression.
(0.03 MB DOC)

Text S1 Supporting Materials and Methods.
(0.04 MB DOC)
