# Supplementary figures and images for: A Negative Regulatory Loop between MicroRNA and Hox Gene Controls Posterior Identities in Caenorhabditis elegans
Source: PLoS Genet. 2010 Sep 2;6(9):e1001089. doi: 10.1371/journal.pgen.1001089 (PMC2932687; doi:10.1371/journal.pgen.1001089)

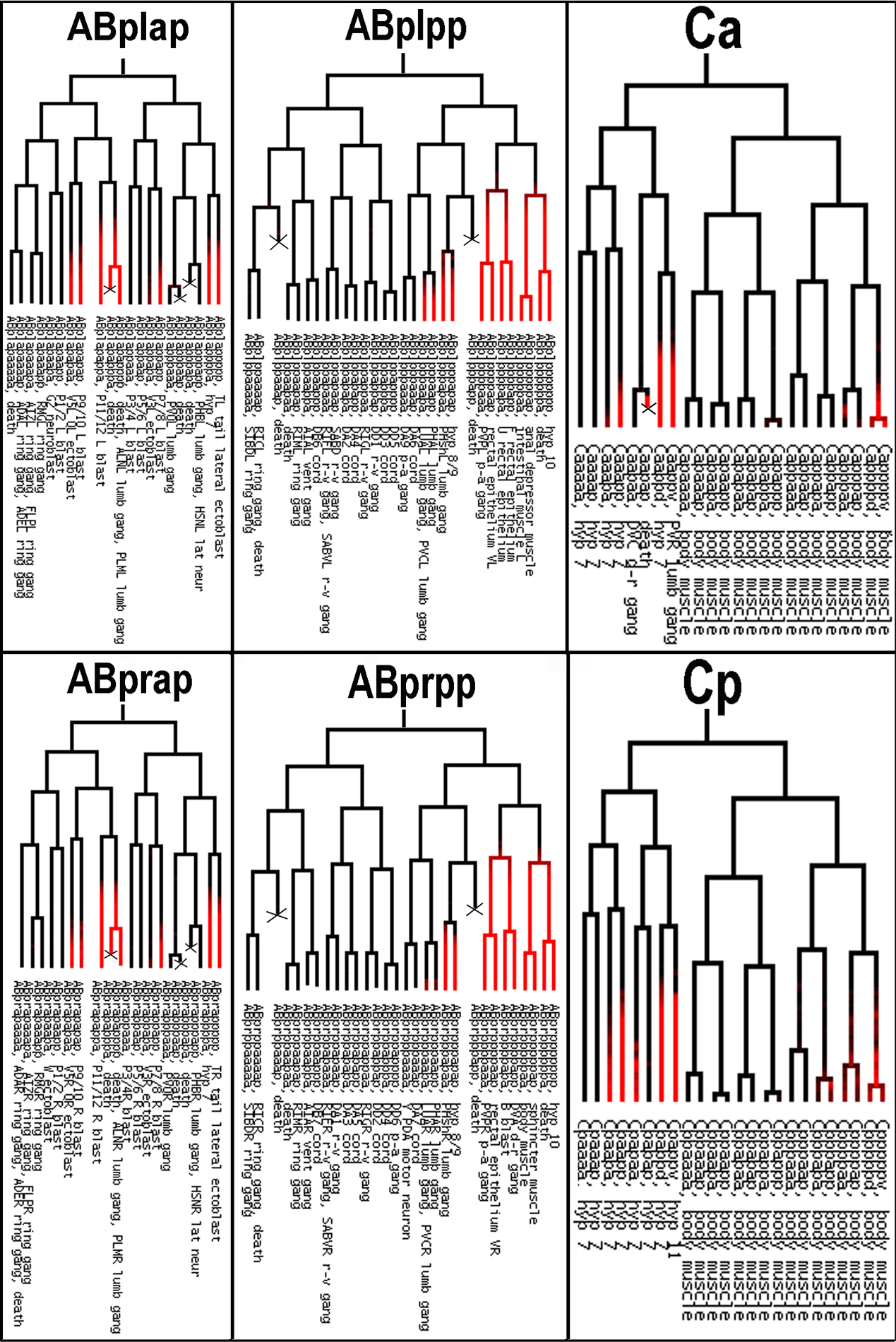

Supplement: Figure S1 — mir-57 shows expression in a variety of cell types in the posterior sublineages. The figure is complementary to the Figure 1 but indicates the terminal cell fates of the expressing sublineages. Vertical bars denote scaled red intensity represented as arbitrary Boyle Unit. (7.26 MB TIF) [file pgen.1001089.s001.tif]

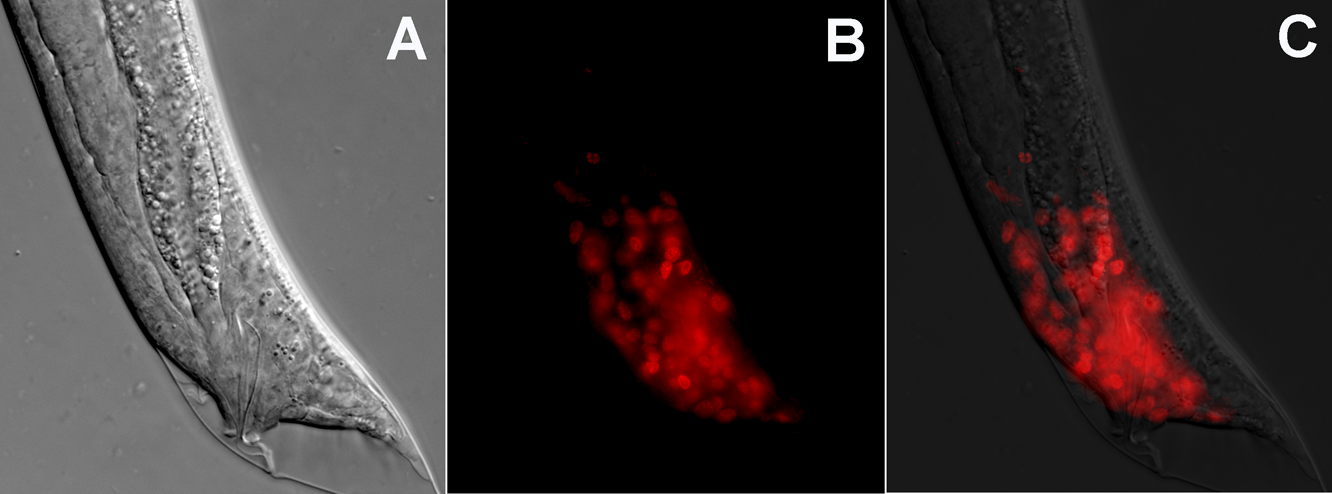

Supplement: Figure S2 — mir-57 expression in adult male tail. (A) DIC; (B) RFP; (C) merged. (2.00 MB TIF) [file pgen.1001089.s002.tif]

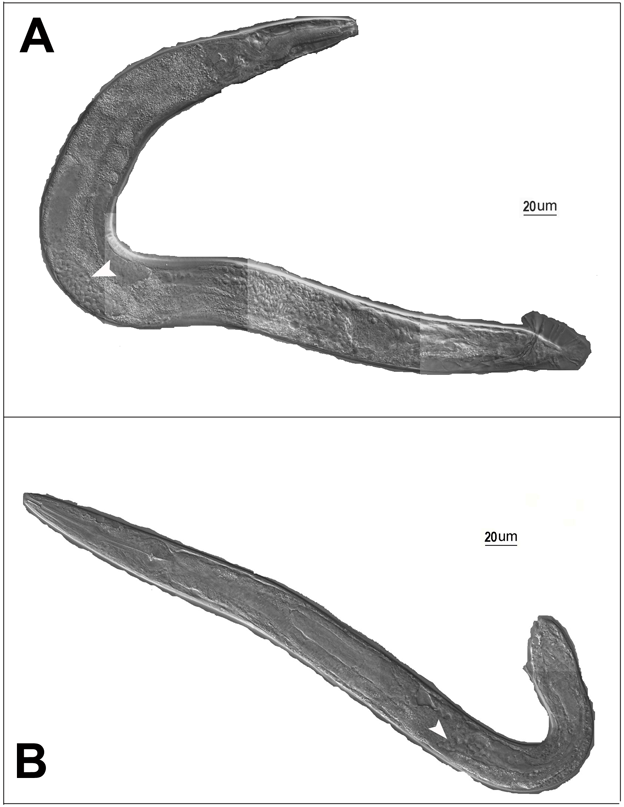

Supplement: Figure S3 — Adult male phenotypes associated with mir-57 overexpression. Note, mir-57 overexpressing animals develop few tail rays and produced only a few sperms (B) compared to N2 (A) animals as indicated by arrow head. (1.54 MB TIF) [file pgen.1001089.s003.tif]

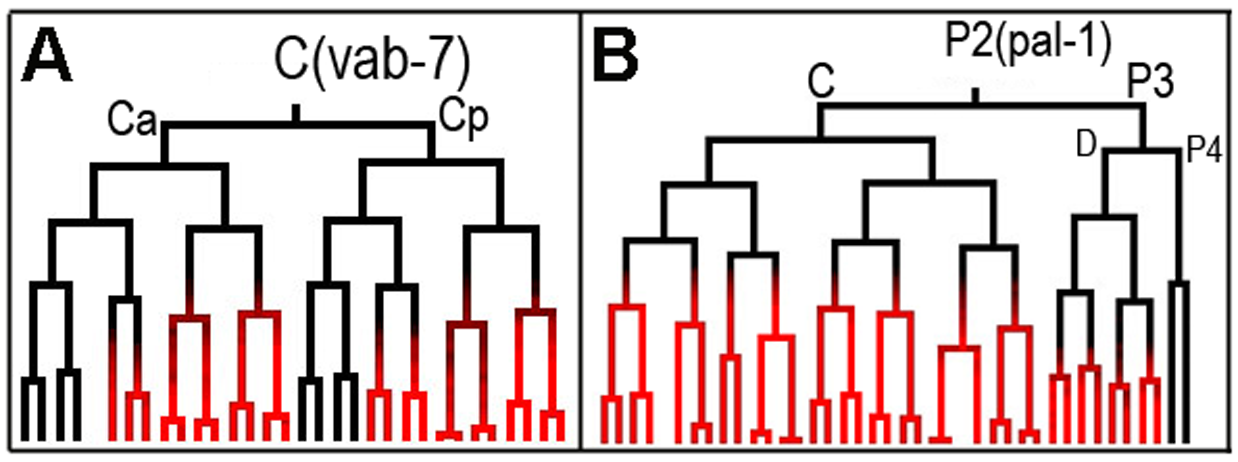

Supplement: Figure S4 — Lineage expression of mCherry reporter driven by vab-7 and pal-1 promoter. (A) vab-7 showed expression in C lineage except for the anterior half of hypodermal sublineages, i.e., Caaaa and Cpaaa. (B) pal-1 showed expression in all P2 sublineage except for germline precursor Z2 and Z4 (not traced as far as other sublineages). Note that all of the reporter-expressing cells are located within the posterior part of embryo (data not shown). (1.74 MB TIF) [file pgen.1001089.s004.tif]

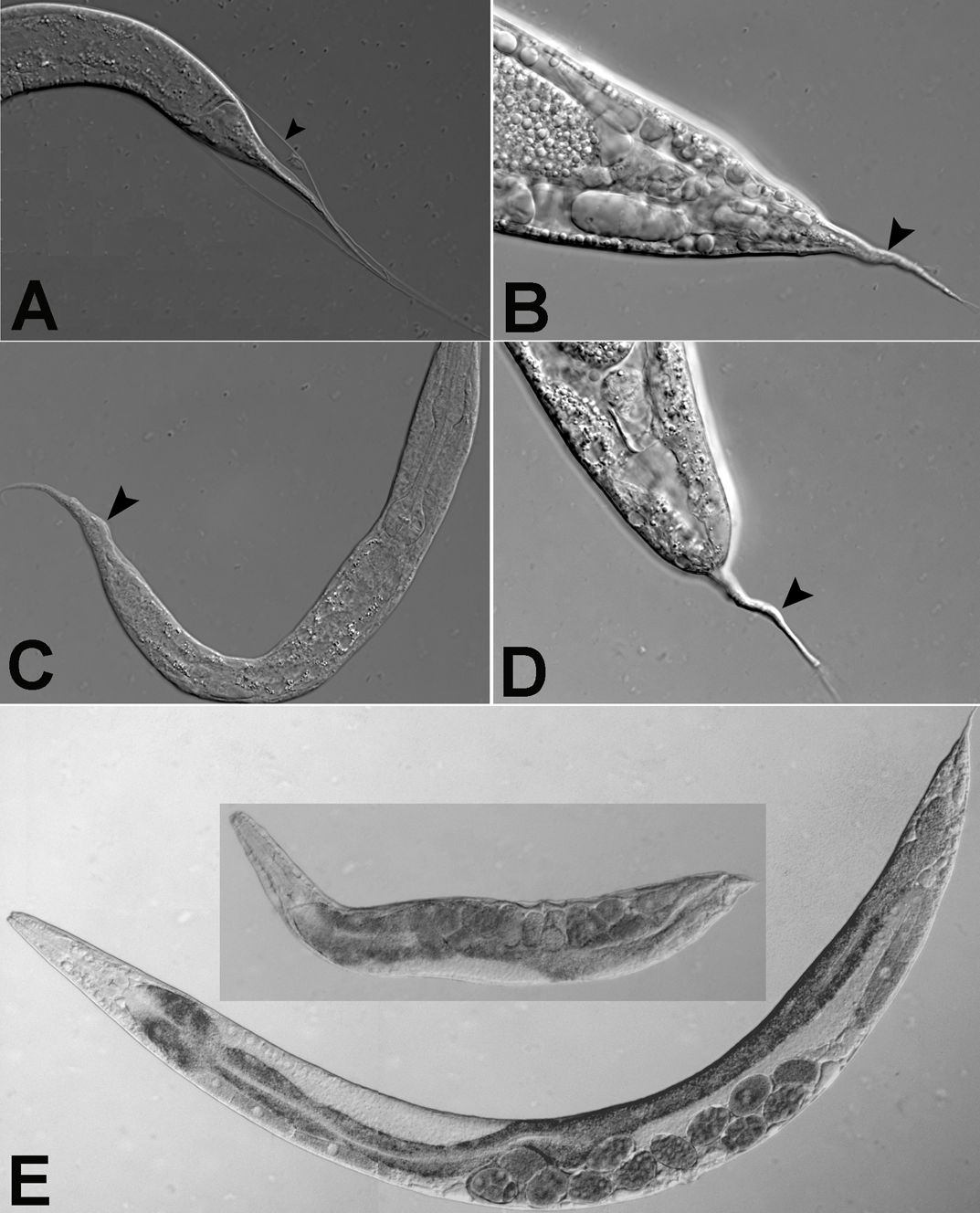

Supplement: Figure S5 — Other posterior defects associated with injection of fusion between vab-7 promoter and mir-57 stem loop sequences. Shown are molting defects (A) and other tail abnormalities (B-D) as well as Dpy (E). (4.30 MB TIF) [file pgen.1001089.s005.tif]

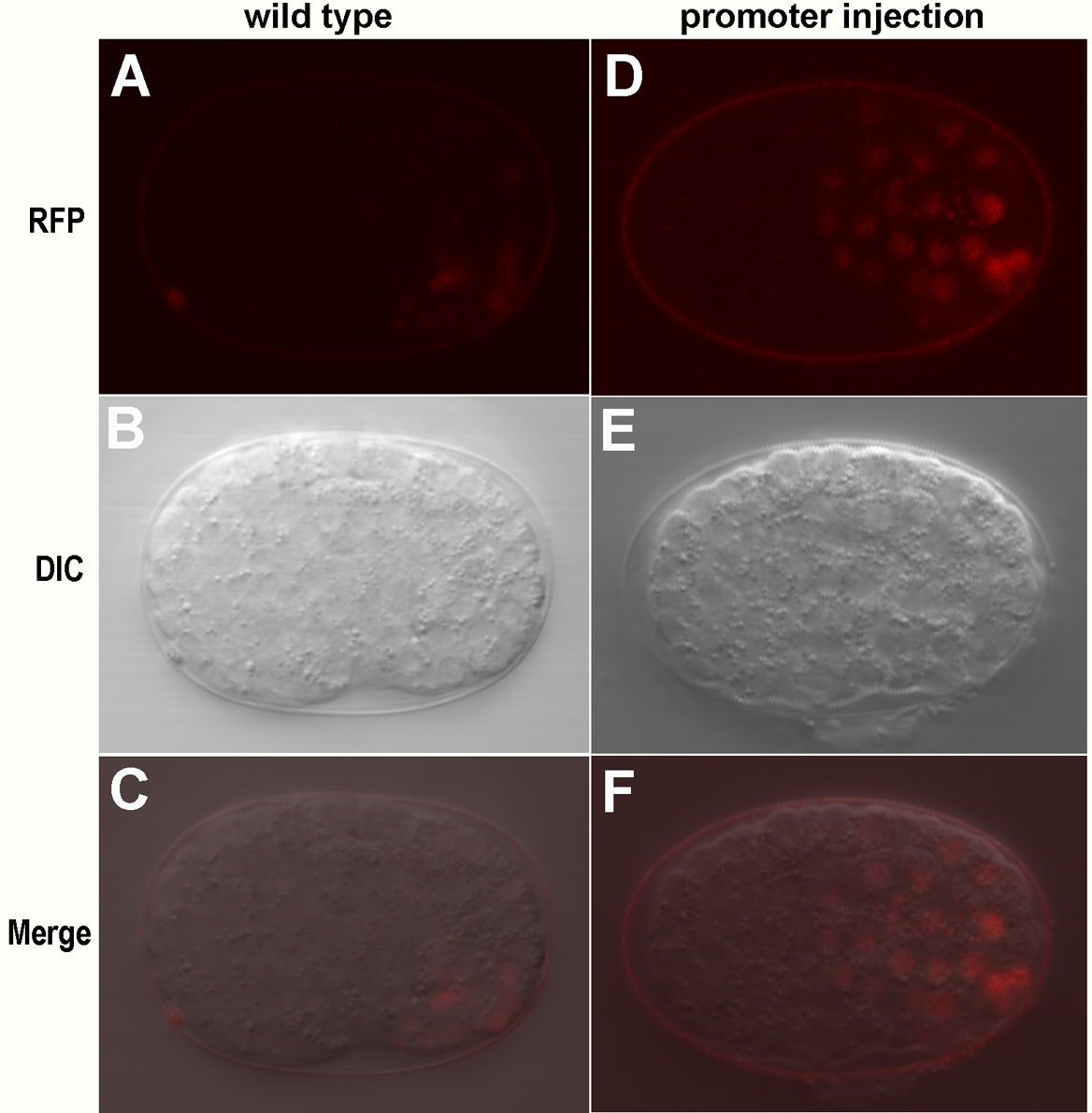

Supplement: Figure S6 — Injection of mir-57 promoters caused earlier onset and ectopic expression (more anterior) of mir-57 in the embryo. An approximately 350 celled embryo was photographed for mir-57 expression in both wild type background animals (A–C) and those carrying the mir-57 promoter array (D–F). The array containing animals were verified by following their tail phenotypes after hatching. (4.20 MB TIF) [file pgen.1001089.s006.tif]

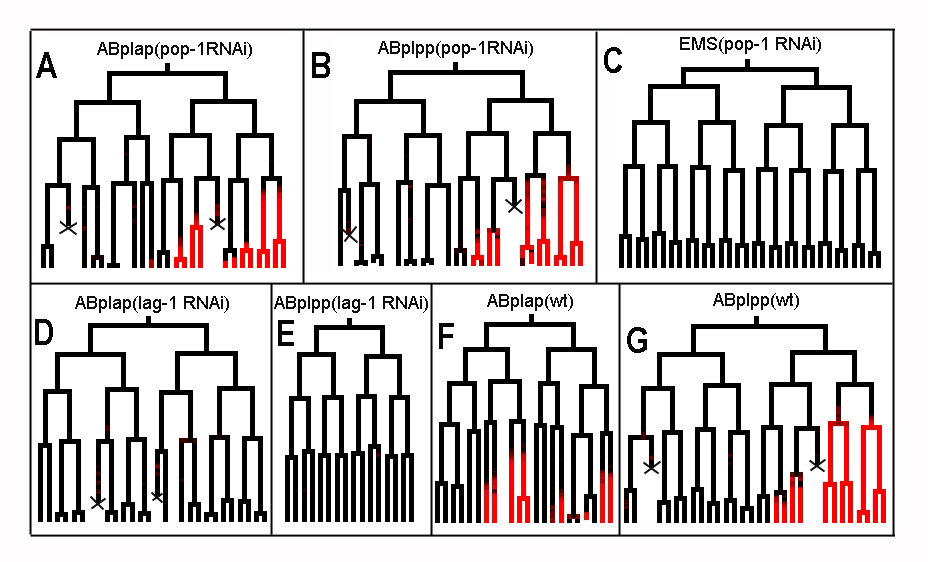

Supplement: Figure S7 — mir-57 expression is dependent on the lineage fate. RNAi against pop-1 produced homeotic lineage fate transformation, i.e., from ABplap into ABplpp (A,F) and MS into E like lineage (C, data not shown) (the later transformation serves as a reference for the RNAi effectiveness) while mir-57 expression and lineage fate remained unchanged in ABplpp (B,G). Note: mir-57 expression in ABplap becomes characteristic of that of ABplpp (A,F,G). RNAi against lag-1 transformed the posterior lineage fates of ABplap and ABplpp into those of anterior ones, i.e., ABalap (D) and ABarpp (E) respectively. mir-57 expression is abolished in both lineages. (1.59 MB TIF) [file pgen.1001089.s007.tif]

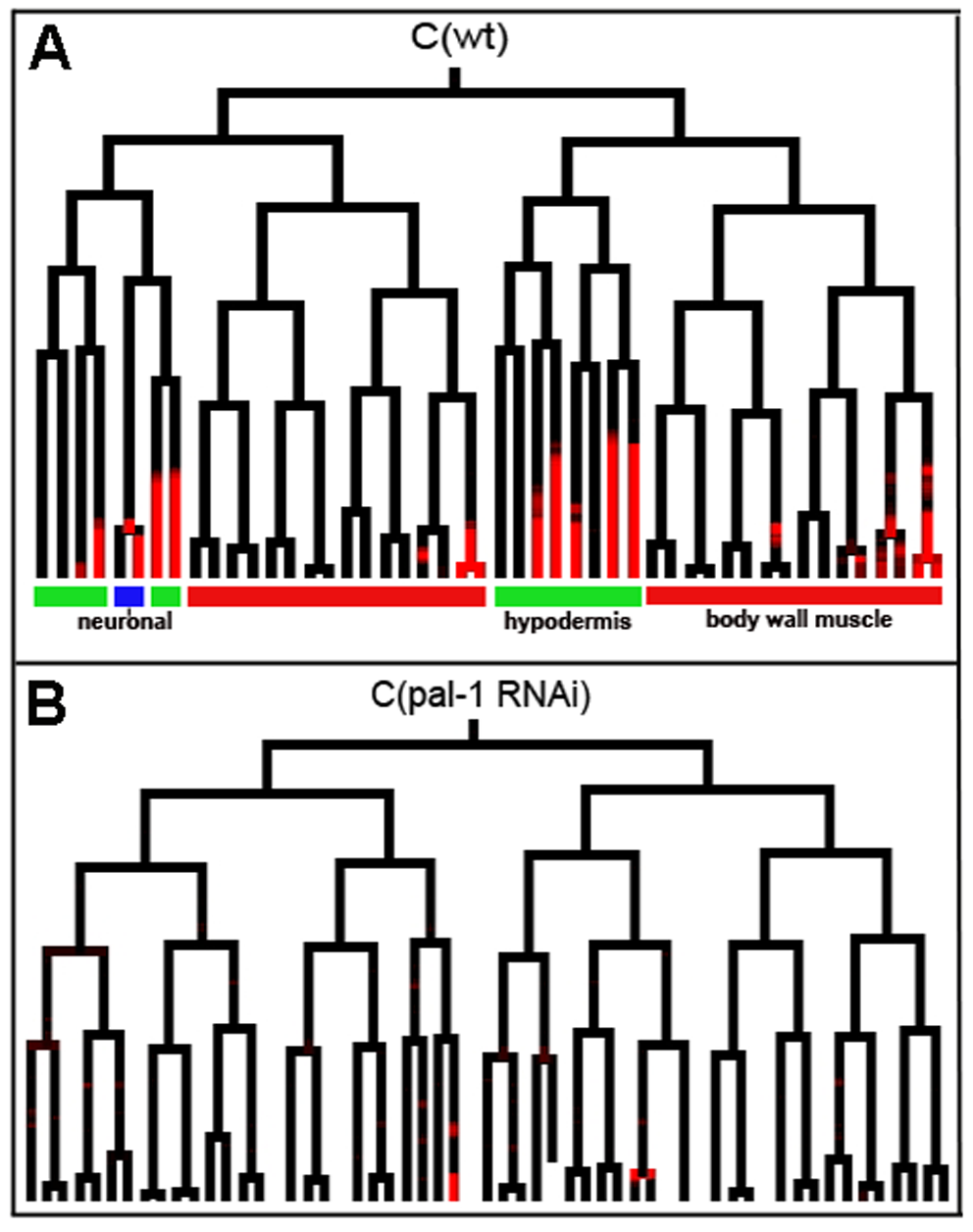

Supplement: Figure S8 — RNAi against pal-1 altered the mir-57 expression in C lineage. Compared to wild type (A), the treatment abolished the fate asymmetry between C derived hypodermis and body wall muscle as well as mir-57 expression (B). (3.77 MB TIF) [file pgen.1001089.s008.tif]
