# Supplementary material for: A Negative Regulatory Loop between MicroRNA and Hox Gene Controls Posterior Identities in Caenorhabditis elegans
Source: PLoS Genet. 2010 Sep 2;6(9):e1001089. doi: 10.1371/journal.pgen.1001089 (PMC2932687; doi:10.1371/journal.pgen.1001089)
Supplement: Table S1 — Phenotypes of NOB-1 overexpression. (0.03 MB DOC) [file pgen.1001089.s010.doc]

**Table S1**. Phenotypes of NOB-1 overexpression

| **Genotype** | **N2 [*rol-6*(+); NOB-1::GFP]** | | | **N2 [*rol-6*(+)]** | | |
| --- | --- | --- | --- | --- | --- | --- |
|  | Emb(%) | Lav(%) | Ste (%) | Emb(%) | Lav(%) | Ste (%) |
| 15 ºC | 1.8(215) | 2.9(233) | 4.0(245) | 1.3(342) | 1.6(237) | 2.1(238) |
| 20 ºC | 2.7(355) | 2.5(352) | 4.1(378) | 1.8(341) | 1.3(239) | 2.4(269) |
| 26 ºC | 5.3(265) | 4.5(323) | 15.8(354) | 2.1(265) | 2.3(354) | 5.5(361) |

Number of worms scored is indicated in parentheses.
