# Supplementary material for: A Negative Regulatory Loop between MicroRNA and Hox Gene Controls Posterior Identities in Caenorhabditis elegans
Source: PLoS Genet. 2010 Sep 2;6(9):e1001089. doi: 10.1371/journal.pgen.1001089 (PMC2932687; doi:10.1371/journal.pgen.1001089)
Supplement: Text S1 — Supporting Materials and Methods. (0.04 MB DOC) [file pgen.1001089.s011.doc]

A Negative Regulatory Loop between MicroRNA and Hox gene Controls Posterior Identities in *Caenorhabditis elegans*

Zhongying Zhao^1^, Thomas J. Boyle^1^, Zongzhi Liu^2*^, John I. Murray^1^, William B. Wood^2^ and Robert H. Waterston^1^

^1^ Department of Genome Sciences, University of Washington, Seattle, Washington, United States of America

^2^ Department of Molecular, Cellular and Developmental Biology, University of Colorado, Boulder, Colorado, United States of America

* Current address: Department of Pathology Informatics, Yale University School of Medicine, New Haven, Connecticut, United States of America

**Supporting Materials and Methods**

**Expression profiling of *vab-7* and *pal-1***

Expression profile of *vab-7* and *pal-1* were generated in the similar way as that for *mir-57* expression profiling. A 3732 and 2101 bp promoter regions were PCR amplified and cloned into pJM20 upstream of his-24 coding region for *vab-7* and *pal-1* using *Avr*II and *Xm*aI or *Avr*II and AgeI sites respectively. The plasmids were bombarded into *unc-119 (ed3)* animals. The integrated strains were crossed into RW10029 to produce lineagable strains for expression profiling assay as that for *­mir-57*.

**Native NOB-1 expression in the presence and absence of *mir-57***

To build native NOB-1 expressing constructs with its 3’ UTR, nob-1 promoter and its coding region were amplified from genomic DNA with the primers listed below. The resulting fragment (about 6.7 kb in size) was cut with NruI and AvrII and ligated with pZZ44 (modified pZZ1 but with nob-1b 3’ UTR) cut with SmaI and AvrII to give rise to pZZ46. The resulting construct was sequenced and used to generate stable transgenic lines using bombardment. Two independent transgenic lines were recovered and both show similar expression patterns (data not shown). The two lines were crossed into *mir-57* deletion strain VC347 and genotyped by single worm PCR. Forty two animals from each line were used for expression assay. Quantification of tail expression was performed in the same way as that used for Figure 8.

**Characterization of embryonic phenotypes of *mir-57* overexpression**

To examine the ectopic expression of *mir-57* after the injection of its promoter sequence, the 2260 bp promoter was co-injected with pRF4 into RW10048 to produce multiple transgenic lines with the concentrations of 20 and 100 ng/µl respectively. The embryos from the roller animals of independent lines were screened for ectopic expression (earlier onset or more anterior expression). The candidate embryos with ectopic *mir-57* expression were recovered from the slides after the images were taken and allowed to hatch and grow for 2 days to check whether they were array containing animals (in this case, roller animals). A total of 12 candidate embryos were used for such recovery and all of them developed into rolling animals (data not shown).

**Characterization of NOB-1 overexpression**

NOB-1::GFP protein fusion construct was shown to be able to rescue *nob-1­* ct230 allele (Wood et al., unpublished data). The construct was co-injected with pRF4(rol-6d) into N2 at 10 and 100 ng/µl to generate three independent lines. Three independent lines were also produced with 100 ng/µl pRF4 only. The following phenotypes were scored at 15°C, 20°C and 26°C respectively for 32 transgenic animals of each line with and without pZZ46: Emb (embryonic lethality), Lva (larva arrest), Ste (sterility).

**RNAi against *pal-1***

RNAi against *pal-1* were done by microinjection with the primers derived from Wormbase (WS203). The RNAi produced 100% embryonic lethality. The embryos were taken for imaging 16 hours after the injection.

All of the post embryonic pictures were taken using a ZEISS Axioplan 2 compound microscope equipped with AxioCamHR camera using a 63X objective lens and those for embryonic ones using a ZEISS LSM510 confocal microscope.

Primers used for transgene expression, overexpression, mutagenesis, target validation (references to Table 2 & Figure 2):

1. primers used for profiling gene expression by automatic lineaging:

*mir-57*-A-his-24: aaaaatgttcccgattgtgtaaa

*mir-57*-B-his-24: gagttcacatacctttttgaatatcat

for *mir-57* overexpression,

*mir-57* genomic fragment from -2260 to 234 relative to its 5’ mature sequences:

*mir-57*-L: gattgtgtaaagcgaggctcat

*mir-57*-R: agacgtgatttcttggctcc

for *mir-57* promoter from -2260 to -63 relative to its 5’ mature sequences:

*mir-57*-L: aatgttcccgattgtgtaaagc

*mir-57*-R: tttt agacgagcatgtcgatgagtt

for heterogenous overexpression driven by *vab-7* promoter from -3664 to 36 relative to its translational start. The *mir-57* fragment was derived from -50 to 223 bp relative to its 5’ mature sequences.

*mir-57*_overexpresion_C: tgttcatgatgatattcaaaaagg

*mir-57*_overexpresion_D: aaaaagtgttgctatttcatacct

*mir-57*_overexpresion_DD:ctatttcatacctaattaatgtcga

vab-7_A: cggtccttttgtctccattt

vab-7_B: cctttttgaatatcatcatgaacaaaccccaataagggactcaat

vab-7_AA: cttttgtctccatttttactac

2. mutagenesis of *mir-57*:

vab-7-mir57-mutagenesis-A: cggtccttttgtctccattt

vab-7-mir57-mutagenesis-B: actcCcatgCtcAgactcGt gacgagcatgtcgatgagtt

vab-7-mir57-mutagenesis-C: aCgagtcTgaGcatgGgagt

vab-7-mir57-mutagenesis-D: aaaaagtgttgctatttcatacctaat

3. site directed removal of LAG-1 site:

lag-1-A-full: aaaaatgttcccgattgtgtaaa

lag-1-B-full: accctcactaaagggaacaaaag

lag-1-deletion-B:

CTTTTTGAATATCATCATGAACAGAATAgaataaaagaagaagttcagaatagagg

his-24-wcherry-868C:tattctgttcatgatgatattcaaaaag

his-24-wcherry-868D:cgcaattaaccctcactaaagg

4. target validation:

mir57-his24-A: aaatgcattccgaaaacacta

mir57-his-24-B: agcgaattccaaagcttCTTA

Ca: TAAGaagctttggaattcgcttgtgaatttttatctaatgtcctaatg

Da: atcatgattaGGGCCCcttttgcctcctttttgcac

Cb: TAAGaagctttggaattcgctgcgattgatcaactgattttctc

Db: atcatgattaGGGCCCcacgtacagagaaaaacgaaaca

Ad: gcgattgatcaactgattttctc

Bd: cgcacaaaaattatgaaattga

AAd: TAAGaagctttggaattcgctgatcaactgattttctctatttacaca

Cd: tcaatttcataatttttgtgcgtgtttttagcccattcttcg

Dd: tcgacggaggagcaagtagt

DDd: atcatgattaGGGCCCagcaagtagtacaggaagtacagga

5. NOB-1 genomic fragment

Nob-1-protein-L: atcgatgcacTCGCGAttgaaaagtttcattctgattttcc

Nob-1-protein-R: atcgatgcacCCTAGG**A**taaaaattcacatttatgatctctagactttc
